# Incorporating pathological gait into patient-specific finite element models of the haemophilic ankle

**DOI:** 10.1007/s10237-024-01857-z

**Published:** 2024-05-20

**Authors:** Harriet G. Talbott, Richard A. Wilkins, Claire L. Brockett, Marlène Mengoni

**Affiliations:** 1 Institute of Medical and Biological Engineering, School of Mechanical Engineering, University of Leeds, Leeds, LS2 9JT UK; 2 School of Engineering, University of Hull, Hull, HU6 7RX UK; 3 NIHR Leeds BRC, University of Leeds, Leeds, UK; 4 Leeds Teaching Hospitals NHS Trust, Leeds, UK; 5 Department of Mechanical Engineering, Insigneo Institute, University of Sheffield, Sheffield, UK

**Keywords:** Tibiotalar, Patient specific, Biomechanics, Finite element analysis, Contact mechanics, Haemophilia

## Abstract

Haemarthrosis is an inherent clinical feature of haemophilia, a disease characterised by an absence or reduction in clotting proteins. Patients with severe haemophilia experience joint bleeding leading to blood-induced ankle arthropathy (haemarthropathy). Altered biomechanics of the ankle have been reported in people with haemophilia; however, the consequence of this on joint health is little understood. The aim of this study was to assess the changes in joint contact due to haemophilia disease-specific gait features using patient-specific modelling, to better understand the link between biomechanics and joint outcomes. Four, image-based, finite element models of haemophilic ankles were simulated through consecutive events in the stance phase of gait, using both patient-specific and healthy control group (n = 36) biomechanical inputs. One healthy control FE model was simulated through the healthy control stance phase of the gait cycle for a point of comparison. The method developed allowed cartilage contact mechanics to be assessed throughout the loading phase of the gait cycle. This showed areas of increased contact pressure in the medial and lateral regions of the talar dome, which may be linked to collapse in these regions. This method may allow the relationship between structure and function in the tibiotalar joint to be better understood.

## Introduction

Haemophilia is an x-linked recessive genetic disorder, where a reduction or absence of coagulation factors leads to spontaneous and traumatic bleeding. Patients with severe haemophilia and moderates with a more severe bleeding phenotype are the most at risk of bleeding; 80% of which occur in the musculoskeletal system (Kasper 2000; Rodriguez-Merchan 2010). Whilst treatment with replacement clotting factor and non-factor treatments significantly reduce the risk of bleeding, patients still report bleeding (Wilkins et al. 2022). A single significant or repeated minor haemarthrosis can lead to joint degeneration known as haemarthropathy. Progressive in nature, this life-long inherent clinical feature is common in people with haemophilia (PwH), with end-stage haemarthropathy occurring as early as the third decade of life (Rodriguez-Merchan 2010). Bleeding most commonly affects the knees, elbows and ankles, with reports of similar bleed rates. The ankle is however disproportionately affected by haemarthropathy (Wilkins et al. 2022), leading to changes in the function and structure of the joint. It is unclear why the ankle is the most affected, a plausible cause is that during activities of daily living, the ankle is exposed to greater compressive and shear stress than the other affected joints. Changes in ankle structure, including flattening of the talar dome, and overgrowth of the distal tibial epiphysis (Gamble et al. 1991; Lundin et al. 2012; Talbott et al. 2021), and function such as loss of range of motion (RoM) (Lobet et al. 2013; Soucie et al. 2004) lead to alteration in gait patterns associated with both the morphology of the haemophilic ankle (Jelbert et al. 2009; MacNicol and Ludlam 1999), and pain-related adaptations (Lobet et al. 2012).

Finite element (FE) models of the natural foot and ankle have previously been used in combination with gait analysis using quasi-static models (Akrami et al. 2018; Anderson et al. 2016; Bae et al. 2015; Chen et al. 2015; Xu et al. 2016; Yu et al. 2013) or, less frequently, dynamic models (Chen et al. 2019; Mo et al. 2022). When considering injured joints, or damaged tissue, these have mostly used a healthy gait profile, rather than diseased characteristics. They have also primarily been whole foot and ankle models, and frequently considered plantar pressure outputs, rather than intraarticular stresses (Talbott et al. 2023). However, understanding how the contact mechanics of the ankle joint change throughout the gait cycle may be pertinent to improving understanding of the adapted gait in PwH. Developing a method to investigate the consequence of patient-specific biomechanical features on image-based FE models would give the opportunity to better understand the potential link with disease progression. In PwH, the relationship between the gait adaptations and the abnormal contact mechanics is important to understand, given the association between contact pressures and the onset of a joint bleed (Buckwalter and Saltzman 1999).

The aim of this work was to incorporate patient-specific gait profiles into image-based FE models to assess the changes in joint contact mechanics due to pathological gait features of the haemophilic ankle.

## Methods

### Data acquisition

T1 weighted clinical MRI data were acquired for four haemophilic ankles, and one healthy sex matched control ankle under local ethical approval (University ethical approval MEEC18-022) following informed consent from participants to use both imaging and biomechanical data (University ethical approval MEEC20-008). In-shoe biomechanical data were also captured for the four haemophilic ankles (UK national ethical approval R&D number: RR19/125282, IRAS code:262181), and 36 healthy control participants (University ethical approval MREC16-087).

### Biomechanical data capture and processing

Each participant in the haemophilia and control group undertook one gait analysis session at a self-selected speed along a 10 m walkway. Lower limb kinematic and kinetic data were captured at Chapel Allerton Hospital (Leeds, UK) in a clinical setting using a 10 camera Vicon system (Vicon MX, Oxford Metrics, UK) at a sampling rate of 100 Hz, with two integrated force plates (AMTI, Watertown, MA) captured at 1000 Hz. The CAST marker set (Benedetti et al. 1998; Cappozzo et al. 1995) was used to track the kinetic and kinematic segments of the lower limb in 6 degrees of freedom. For the haemophilic ankles, the most affected joint was used for analysis—corresponding with the four sets of MRI data. For the healthy control participants, biomechanical data were captured for the dominant limb. All participants had a period of familiarisation prior to data collection of five successful trials.

Kinetic and kinematic in-shoe data for the normal (n = 36) and pathological (n = 4) cases were collected using a bespoke in-shoe single segment kinetic foot model during the stance phase of the gait (Wilkins 2021). Kinetic and kinematic with markers placed in-shoe for the normal cases had excellent correlation with markers placed on the foot, with RMS error of the order of 1% (Wilkins 2021). The ankle joint centre was defined as the midpoint of the malleoli, and 3D printed cluster wands were placed on the foot through 25 mm apertures at the lateral calcaneus and the heads of the first and fifth metatarsals on the dominant or affected foot (Wilkins 2021). The contralateral foot was defined by placing 9 mm reflective markers on the study footwear.

**Fig. 1:**
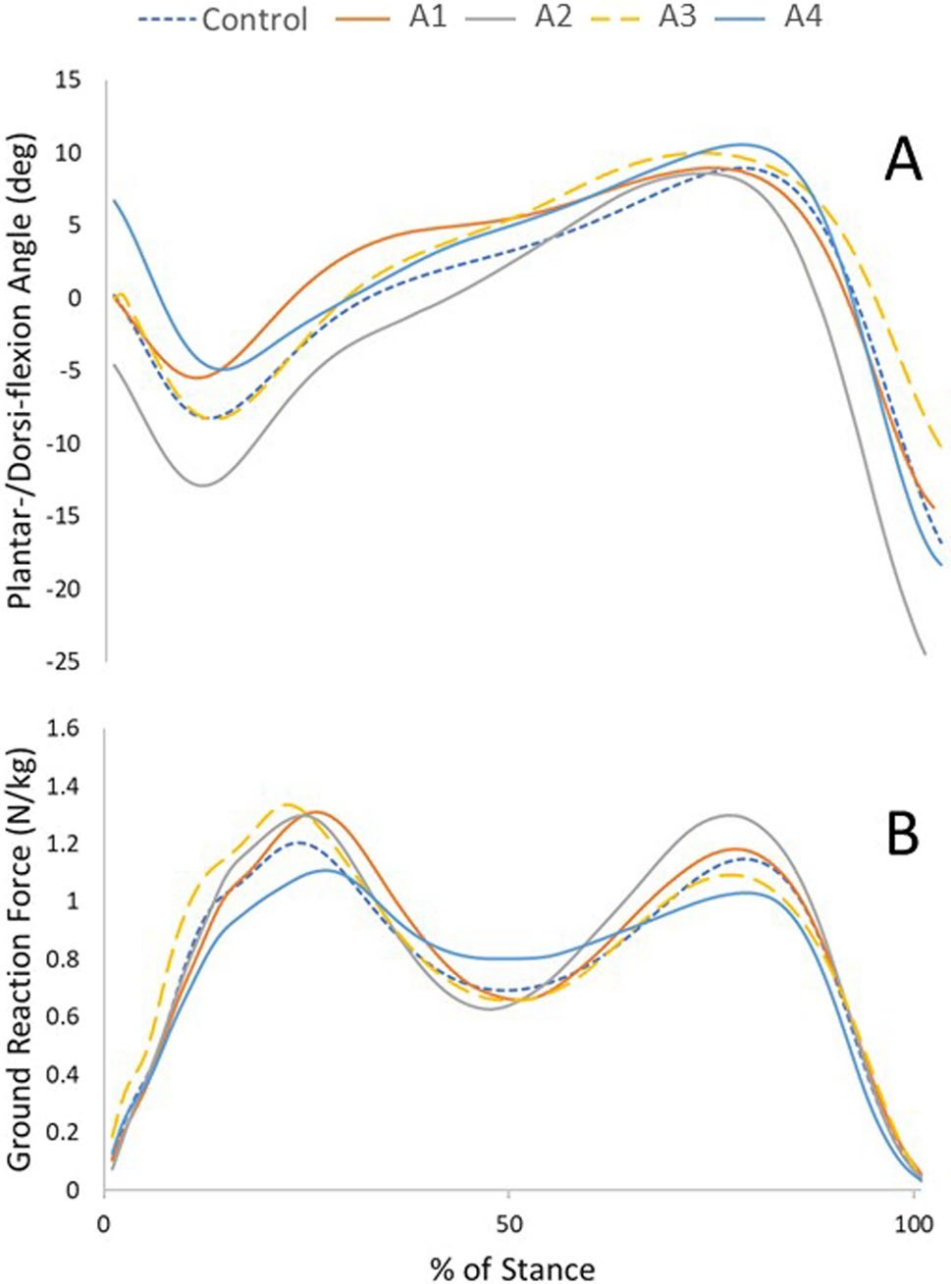
**A**, plantar/dorsiflexion angles, and **B**, ground reaction force through the stance phase of gait for the control group, and the four haemophilic ankles (A1-A4)

Kinetic and kinematic marker trajectories were exported to and analysed with Visual 3D (C-Motion, USA). Kinematic data and ground reaction forces (GRF) were filtered using a Butterworth filter (6 Hz and 25 Hz, respectively) with cut off above 20 N at heel strike and below 20 N for toe off using thresholds from the GRF data (Richards 2008; Robertson and Dowling 2003). Kinetics and kinematics were normalised to 100% of the stance phase of the gait cycle in the sagittal, frontal, and transverse planes. This gave 101 data points over the stance phase of gait (Fig. 1), from which, the five points of interest (heel strike, foot flat, mid stance, heel off and toe off) were selected. Heel strike was defined as initial contact, foot flat at the first peak of plantar flexion (~ 15% stance phase), midstance at minimum vertical GRF (around 50% stance phase), heel off as peak dorsiflexion (~ 80% stance phase), and toe off as the final data point of stance phase.

### Finite element modelling

Patient-specific FE models were simulated in a quasi-dynamic manner, where sequential quasi-static model of up to five events in the gait cycle were modelled. Four versions of the gait cycle were used: the healthy in-shoe gait profile, the healthy in-shoe gait profile altered with two different reductions of RoM thought to be representative of PwH (Gamble et al. 1991), and the patient-specific in-shoe gait.

Processing of five MRI sequences (four heamophilic ankles and one healthy control) to generate segmentation specific meshes of the tibia, talus and their respective articular cartilages was carried out using Simpleware ScanIP (version P-2019, Synopsis Inc., Mountain View, California) using direct thresholding, morphology operations, and slice-by-slice manual correction. The tibia and tibial cartilage were offset by 1 mm, so there was no initial contact between the tibial and talar cartilage in order to enhance mesh quality and associated simulations convergence. All meshes were quadratic tetrahedral meshes, with a minimum edge length of 1 mm following convergence analysis (Talbott and Mengoni 2024). FE meshes were imported into Abaqus CAE (Abaqus, version 2017, Dassault Systèmes, Vélizy-Villacoublay, FR), which was used for the nonlinear FE analysis.

Bone was modelled as a linear homogeneous isotropic material and cartilage as an incompressible isotropic hyper-elastic material (Talbott et al. 2022). The contact between the two cartilage layers was defined as surface-to-surface penalty contact with a coefficient of friction of 0.1 to represent the haemarthritic nature of the cartilage.

Two studies were carried out utilising these five ankle models. Firstly, the effect of and sensitivity to a reduction in RoM, with respect to a healthy gait, was assessed; secondly, patient-specific gait was applied to the respective ankle models and contrasted with a healthy gait profile in the same anatomy. The healthy gait was also applied to the healthy anatomy as a point of comparison.

**Fig. 2:**
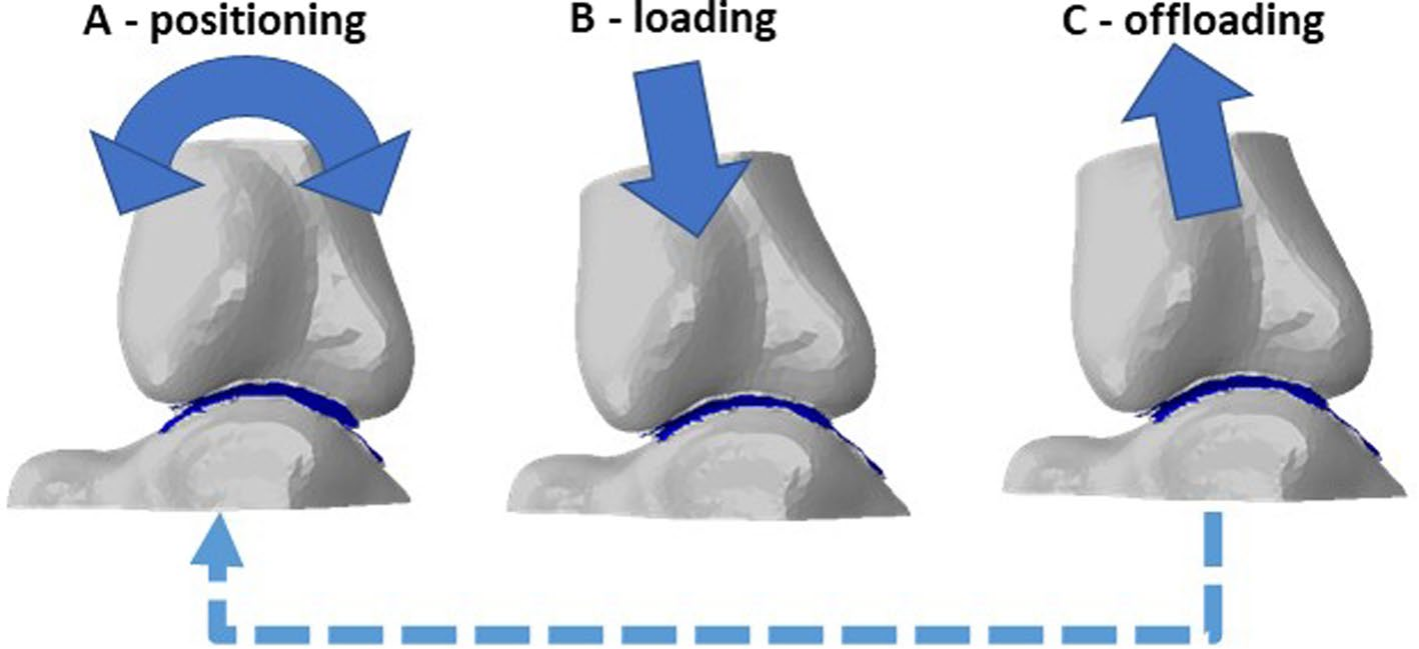
Example of steps progress through **A**, positioning with rotations and translations **B,** loading of the tibia to force F, and **C,** offloading of the tibia to zero force to achieve each of the points in stance phase. Process was repeated for each event of stance phase within one simulation (dashed arrow)

In both studies, the same method was utilised to position and load the model for each of the events simulated in the stance phase. Rotations and translations (Fig. 2A) of the tibia were sequentially applied to move the joint into a position representing the event in the gait cycle. A load was then applied axially to the tibia (Fig. 2B). In the patient-specific models, the vertical component of the GRF at that point in stance phase was used as an upper estimate of the load through the joint, whilst in the reduction in RoM models, no adjustment was made to the load with respect to the healthy gait data. This process was repeated, following the unloading of the model to remove any contact between the joint surfaces in the rotation (Fig. 2C). The simulation cycled through these processes in continuous steps to mimic the events of interest in the stance phase.

The loads and boundary conditions were applied to the tibia through a kinematic coupling with a reference point at the approximate centre of rotation of the tibiotalar joint. The coordinate system was set with the z-axis running axially through the tibia and following its rotation, and the x-axis aligned to apply rotations in the sagittal plane.

Examples of the boundary conditions applied in the positioning and loading steps can be seen in Table 1 with a displacement applied to initiate contact, which is required because of the offset applied at segmentation or following each offloading step. Offloading allows relative motion between diseased segmentation specific cartilage where the surface is far from smooth and would otherwise cause convergence issues in computing contact under load. The joint angle varied depending on the gait data input to represent plantar/dorsiflexion. Boundary conditions represented the stabilising effect of ankle ligaments. In the loading step, rotation was fixed at reached position to ensure; no additional tibial rotation was allowed. The loads and boundary conditions were set to follow nodal rotation, so when the tibia moved to each new position, the loads were applied in the axis of the tibia.

**Table 1:** Example boundary and loading conditions for the rotation and loading steps, where A is displacement required to make contact between tibial and talar cartilage surfaces, θ is the joint angle in the sagittal plane, in radians, and F is the force calculated based on patient weight. Loads in the tibial axis direction follow the nodal rotation. The z direction is defined as following the axis of the tibia; rotations around the x-axis are plantar/dorsiflexion rotations

Step	Displacement			Rotation			Load		
	**x**	**y**	**z**	**x**	**y**	**z**	**x**	**y**	**z**
Positioning	0	0	-A	θ	0	0	free	free	free
Loading	0	0	free	Fixed at current position			free	free	0 to F
Offloading	0	0	free	Fixed at current position			free	free	F to 0

### Study 1: Effect and sensitivity to a generic reduction in RoM

The aim of the first study was to understand if there was a change in contact mechanics with reduced RoM. Varying degrees of loss of RoM have been cited for PwH, up to 80% reductions (Gamble et al. 1991) and mainly in plantar/dorsiflexion (Soucie et al. 2004). To investigate this, each of the four haemophilic ankle model was simulated through three points in the stance phase of gait (max plantarflexion, mid stance, max dorsiflexion). Besides healthy values of maximum plantar/dorsiflexion (14 degrees), two generic reductions in plantarflexion and dorsiflexion were applied (50% and 80%). The same generic percentage patient body weight (Talbott et al. 2022) was used for all simulations, to ensure the reduction in RoM was being treated as an independent variable.

Peak and mean contact pressures were recorded alongside the contact area for the dorsiflexion and plantarflexion on each cartilage surface of each ankle. The differences between the healthy gait case and 50% reduced joint angle, and the healthy gait case and 80% reduced joint angle were calculated for the four haemophilic ankles.

### Study 2: Patient-specific biomechanical modelling

The joint angles calculated from the in-shoe biomechanical data for the four haemophilic ankles (Table 2) did not all show the large reductions in RoM reported in literature. Therefore, the four haemophilic FE models were modified to consider the influence of patient-specific gait, and a healthy control model was developed using the same method to give a healthy point of comparison.

**Table 2:** Joint Angles (degrees) at each point simulated in the gait cycle for data extracted in this study (control and four PwH ankles, all with markers in-shoe) and compared to literature for a healthy cohort (the variation in the data extracted from the literature is about 10 degrees, markers on foot)

	Heel Strike	Foot Flat	Mid Stance	Heel Off	Toe Off
Control	0.2	− 8.2	3.4	9.0	− 16.8
Ankle 1	0.7	− 5.5	5.6	9.0	− 14.4
Ankle 2	− 3.2	− 12.6	1.4	8.6	− 24.4
Ankle 3	0.3	− 8.4	6.0	10.0	− 11.0
Ankle 4	6.7	− 4.9	4.8	10.6	− 18.3
Leardini et al. 2014	− 3.5	− 7.5	3	9.5	− 8.5

Each FE model was simulated through five points in the stance phase of gait (heel strike, foot flat, mid stance, heel off and toe off) using both patient-specific biomechanical data, and an average of the 36 healthy control participants captured as control biomechanical data. The healthy control geometry data were also modelled, using the average control biomechanical data as the MR imaging data were not from a participant of the healthy control biomechanical analysis group.

The vertical component of the GRF from the biomechanical data (Fig. 1) was used as the load applied to each ankle model in the loading step, for each point in the gait cycle (Table 3). When simulating the haemophilic ankle models through the control gait, the loading conditions reflected the average control GRF, corrected for patient bodyweight.

**Table 3:** Load (N) applied to gait model for each haemophilic ankle, calculated from GRF

	Heel Strike	Foot Flat	Mid Stance	Heel Off	Toe Off
Control	79.3	678.7	510.3	837.8	30.1
Ankle 1	73.8	598.1	465.8	813.3	37.7
Ankle 2	53.4	658.5	460.8	938.1	28.2
Ankle 3	125.7	720.5	442.0	705.2	22.2
Ankle 4	117.6	806.0	740.2	946.0	28.4

Nodal values of the bones von Mises stress field and the contact pressure fields on each cartilage surface were considered in Kolmogorov–Smirnov paired nonparametric tests for three of the four haemophilic ankles in intra-subject comparisons. The fourth haemophilic ankle did not simulate through the healthy RoM, as it was unable to rotate to the extremes of plantar-/dorsiflexion due to diseased anatomy. Assessing the difference in outputs between patient-specific gait and control biomechanical input data at the first and second ground reaction force peaks, as well as the trough at mid stance, gave nine paired tests for each of tibial and talar cartilage contact pressure distributions, and tibial and talar von Mises stress distribution.

Intra-subject analysis was also carried out qualitatively for each of the five models, assessing how the contact pressures and areas moved through stance phase progression. The inter-subject analysis followed a similar methodology, to visualise the difference between the four haemophilic ankles and a healthy control ankle model at each stage of stance phase.

## Results

All data associated with the FE models in this paper are available openly (Talbott and Mengoni 2024).

### Study 1: Effect and sensitivity to a generic reduction in RoM

**Fig. 3:**
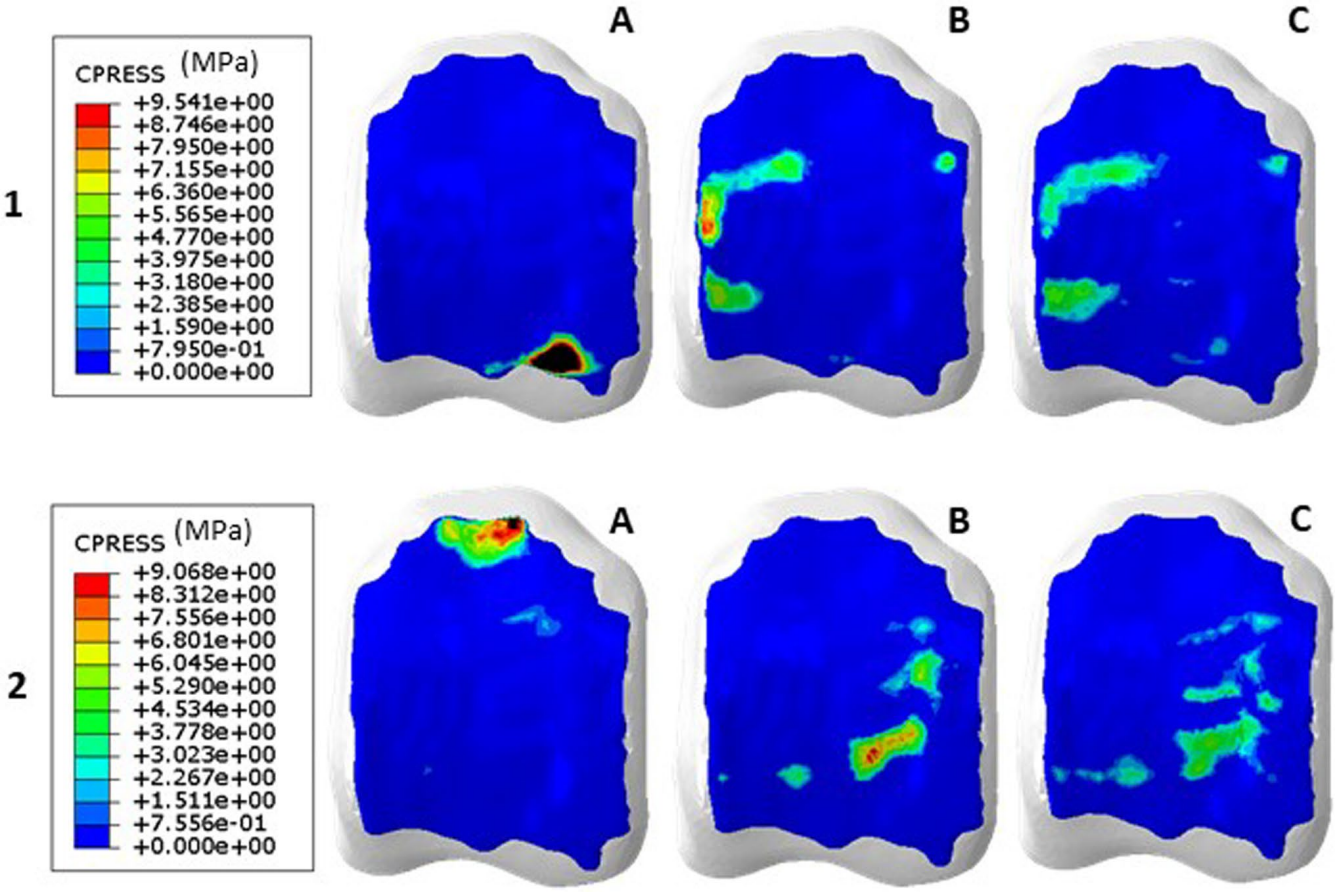
Altered contact distribution for 1) dorsiflexion, and 2) plantarflexion, between **A**, 14 degrees **B,** 7 degrees (50% reduction) and **C**, 2.8 degrees (80% reduction) in hameophilic ankle 2

Reductions in average contact pressures were seen in both the 50% and 80% reduced joint angles at both plantarflexion and dorsiflexion of the haemophilic ankles (Table 4). Changes in peak and mean contact pressures were not systematic at dorsiflexion, with a 40% increase in mean contact pressure in one ankle, whilst others decreased by 10 to 65%, at 50% reduction in RoM. Contact area increased with reductions in RoM; a redistribution effect was also seen at the reduced joint angles (Fig. 3), with similar areas in contact for both reduced RoM conditions which differed from the healthy case.

**Table 4:** Average percentage changes in contact pressure (peak and mean), and contact area between 14 degrees joint angle and 7 degrees (50% reduction), and 14 degrees and 2.8 degrees (80% reduction)

	Tibial Cartilage			Talar Cartilage		
	Peak Pressure changes (%)	Mean Pressure changes (%)	Contact Area changes (%)	Peak Pressure changes (%)	Mean Pressure changes (%)	Contact Area changes (%)
Dorsiflexion						
50% reduction	1.8	− 8.0	34.3	− 11.8	− 14.9	32.4
80% reduction	− 4.9	− 17.0	104.4	− 20.1	− 24.4	86.6

Plantarflexion						
50% reduction	− 9.9	− 22.2	37.6	− 7.1	− 14.0	45.9
80% reduction	− 27.1	− 39.7	85.0	− 9.5	− 23.3	105.6

### Study 2: Patient-specific biomechanical modelling

The paired nonparametric tests showed significant differences in that the cartilage contact pressure distribution between the control gait and patient-specific gait for all comparisons on the talus (*p* < 0.005) and in eight of the nine comparisons on the tibia. These differences were not systematic, with both increases and decreases in contact pressures between the patient specific and control gait (Table 5). There was no significant difference between the tibial cartilage outputs at midstance in one ankle (*p* = 0.193). The bone results were a little more variable, with significant differences in von Mises stress distributions in seven of the nine comparisons in the tibia and only two of the tali.

**Table 5:** Mean contact pressure (MPa) in tibial and talar cartilage and mean von Mises stress (MPa) in tibia and talus at foot flat (FF), mid stance (MS) and heel off (HO). Where * represents significant differences (p < 0.005) in paired nonparametric tests

	**A1**			**A2**			**A3**		
**Mean Contact Pressure (MPa)**									
Tibial Cartilage	FF*	MS	HO*	FF*	MS*	HO*	FF*	MS*	HO*
Patient Specific	5.05	3.76	7.47	3.79	2.23	4.41	3.72	1.89	3.63
Control Gait	4.75	4.46	7.65	4.69	1.57	4.31	4.02	2.40	3.60

Talar Cartilage	FF*	MS*	HO*	FF*	MS*	HO*	FF*	MS*	HO*
Patient Specific	5.35	3.07	6.36	5.48	3.59	6.49	5.98	2.75	5.04
Control Gait	4.65	3.56	6.61	7.21	1.97	6.29	6.35	3.26	5.04

**Mean von Mises Stress (MPa)**									
Tibia	FF*	MS*	HO*	FF*	MS*	HO*	FF*	MS	HO
Patient Specific	1.13	0.83	1.66	1.13	1.11	1.82	1.09	0.74	1.37
Control Gait	1.06	0.94	1.59	1.20	0.66	1.82	1.25	0.80	1.30

Talus	FF	MS*	HO*	FF	MS	HO	FF	MS	HO
Patient Specific	2.86	1.97	3.41	1.18	0.95	1.58	0.86	0.59	1.01
Control Gait	2.24	2.18	3.40	1.21	0.77	1.57	0.98	0.60	0.95

**Fig. 4:**
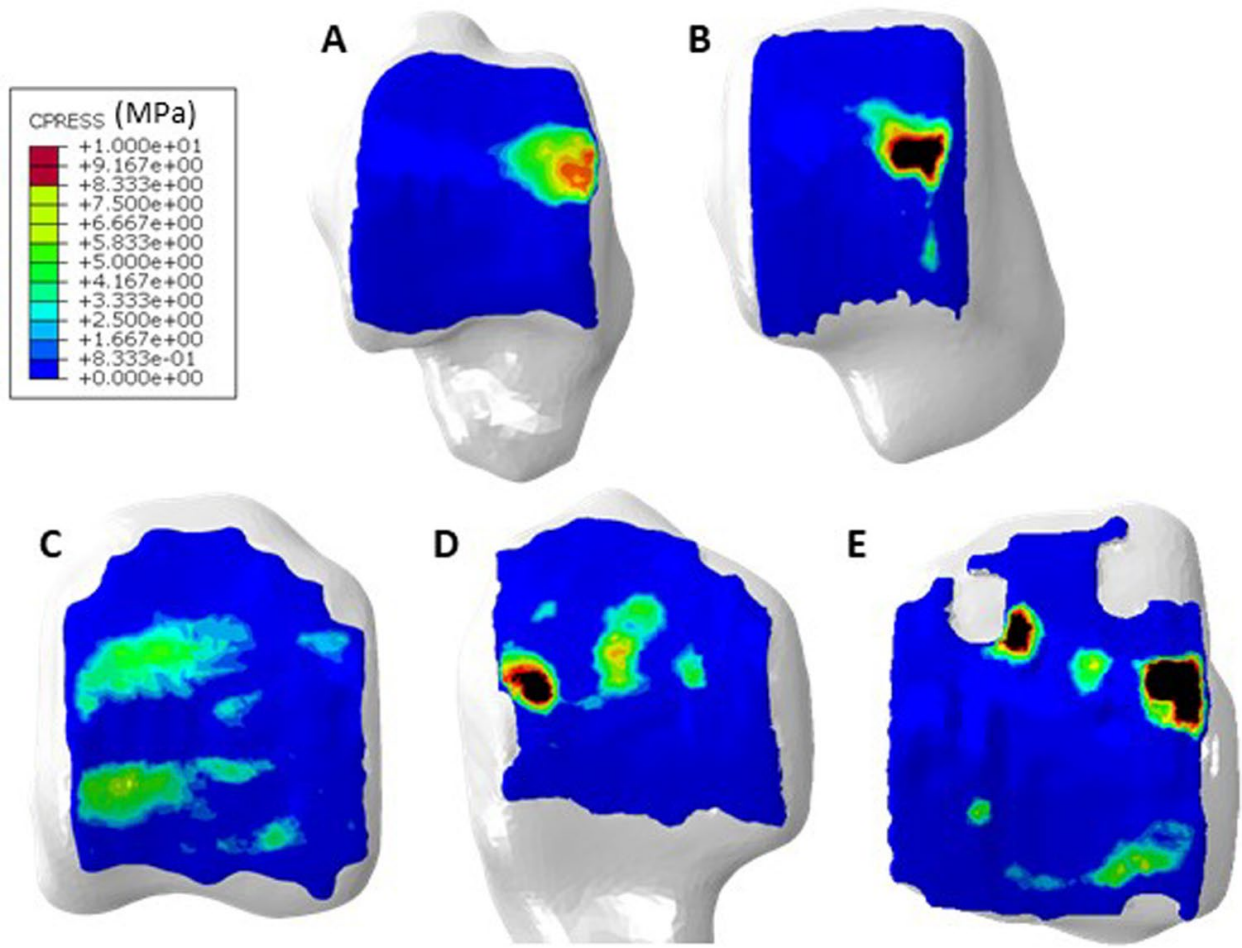
Contact distribution at mid stance in **A**, healthy control **B**, Ankle 1 **C,** Ankle 2 **D,** Ankle 3 and **E,** Ankle 4

**Fig. 5:**
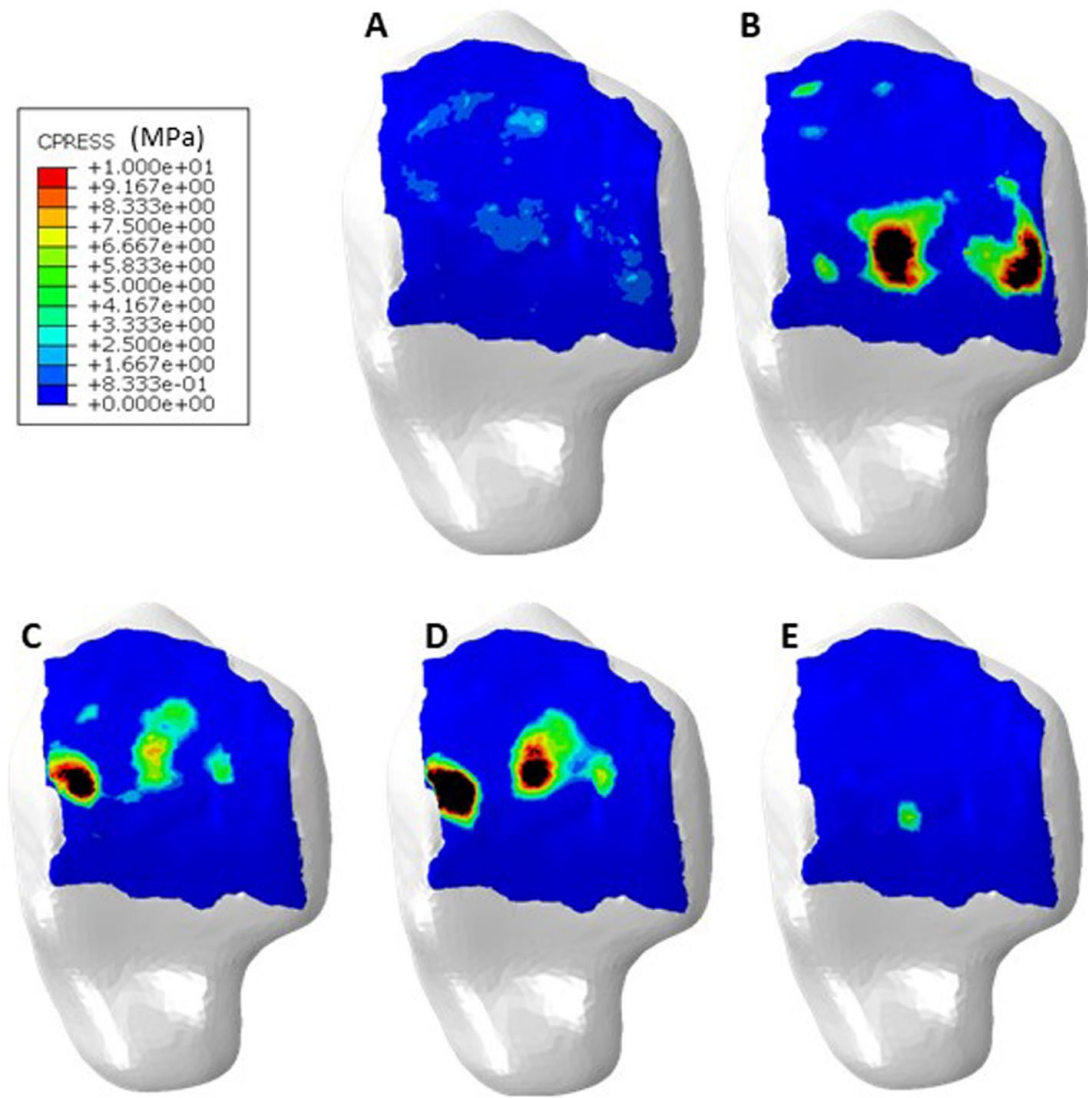
Change in contact distribution between **A**, Heel Strike **B**, Foot Flat **C**, Mid Stance **D**, Heel Off and **E**, Toe Off in haemophilic ankle 3

Contact location as well as contact pressure values at mid stance varied between each of the four haemophilic ankles and with respect to the healthy control one (Fig. 4). The distribution through gait (Fig. 5) differed for each ankle. Peak contact pressures occurred generally in the medial and lateral regions of the joint for the haemophilic ankles, whilst in the control ankle, the areas of contact moved in an anteroposterior direction in the medial/central talus (further examples of talar cartilage contact pressures through stance phase of gait are available in the data associated with this paper (Talbott and Mengoni 2024)).

## Discussion

This study has identified that gait adaptions due to haemophilic arthropathy of the ankle joint are highly variable. Each patient had a slight difference in their walking strategy, with patient-specific adaptation at the ankle progressing through the stance phase of the gait cycle.

In the frontal plane, models of patients in this cohort exhibited greater stresses to the medial and lateral regions of the talus throughout the stance phase of gait, with significant flattening found in the same regions (Talbott et al. 2021). These findings suggest that destabilisation of the joint during periods of haemarthrosis and gradual plantarflexion deformity of the ankle leads to compensatory loading of the medial and lateral talus and not limited to the central talar dome reported previously (Chang et al. 1993, 2001).

Reduction in ankle plantarflexion RoM is commonly reported in the haemophilia, with gradual plantarflexion deformity of the ankle as haemarthropathy progresses (Gamble et al. 1991). However, the reductions in sagittal plane plantar/dorsiflexion RoM in the patient cohort were smaller than reported in patients with multi joint haemarthropathy in a larger cohort of patients with advanced haemarthropathy (Lobet et al. 2012). The results of this study indicate that losses in ankle kinematics may be affected in the later stages of haemarthropathy; however, the small sample size limits inference to the general haemophilia population.

Whilst there is no evidence that biomechanical changes at the ankle joint are a limitation specifically due to morphological differences (Jelbert et al. 2009; MacNicol and Ludlam 1999), this study has however provided an assessment tool for this relationship through the stance phase of gait to be investigated.

The generic reduction in RoM, and paired analysis between patient specific and control gait, was carried out due to both morphology and biomechanical input data being variables in the models. When contrasting the four haemophilic ankles, and healthy control ankle, at midstance, haemophilic ankle 1 and haemophilic ankle 3 had the most similar joint angle input data, yet the contact distributions differed greatly, highlighting the fact that morphology, as well as biomechanical adaptation, impact the contact mechanics throughout the stance phase of gait.

Specific validation of the FE models for this cohort was not performed and conducting such validation with patient with a rare disease would pose ethical challenges. Moreover, little published work is available for in-shoe ankle dynamics to use as comparison point. However, the contact pressure values at foot flat for the healthy ankle are similar to those reported through discrete modelling methods (Benemerito et al. 2020), and the pressure distribution lateral to medial migration during the stance phase was similar to that in other FE studies (Muralidharan et al. 2020), also showing smaller contact area in disease ankles.

This study is limited by the material properties used for all tissues, which were from healthy ankle literature. A relationship between mechanical properties and tissue composition has been reported previously (Kiviranta et al. 2006); therefore, it is unlikely the values used accurately represent the haemarthritic condition. However, such differences are more likely to affect the magnitude than the distribution of forces and contact stresses—meaning these preliminary findings through stance phase still provide valuable information.

As the biomechanical model was a single segment foot model, all plantar/dorsiflexion has been assumed to occur at the tibiotalar joint. This is known to be a simplification; however, it is the most common biomechanical method of modelling ankle kinetics (Cappozzo et al. 1995; Ferrari et al. 2008) and allows for the FE models to be driven directly by loading and boundary conditions, without the need for additional soft tissue structures such as ligaments. The method utilised could be adapted to include subtalar motion through different kinematic couplings. It could also be further enhanced by adding inversion/eversion and internal/external rotation. However, in PwH, the greatest loss of RoM is seen in plantar/dorsiflexion (Soucie et al. 2004) hence the focus on this motion. The method could also be adapted to include the other components of GRF as these are not negligible whereas they have been treated as such in the current models.

The development of this quasi-dynamic model does not take into consideration the effect of inertia, as a dynamic model would. However, it is an improvement on quasi-static models given it takes into consideration the influence of the joint movement into position as part of the simulation. The rigorous development of this method ensured that factors such as the loading/offloading and rotations/translations did not impact the quantitative or qualitative model outputs, allowing for the joint alignment to be included in the simulation. This in turn allows multiple points of stance phase to be considered collectively.

## Conclusion

The method developed in this study allowed diseased and healthy ankles to be simulated through consecutive points in the gait cycle to investigate the influence of generic reductions in range of motion, and of patient-specific biomechanical data. The analysis of these models provides insight into the influence of adapted biomechanics that occur in haemarthropathy on contact mechanics in stance phase of gait. In this patient cohort, it showed a lateral to medial contact pressure migration in the stance phase, with larger stresses in these regions compared to healthy participants. This application could be translated to other conditions, such as post traumatic osteoarthritis where the plantar/dorsiflexion motion is impacted, and provide treatment targets for intervention trials.

## Data Availability

All data associated with the FE models in this paper are available openly (Talbott and Mengoni 2024).
